# Association between Gut Microbiota and Infant’s Temperament in the First Year of Life in a Chinese Birth Cohort

**DOI:** 10.3390/microorganisms8050753

**Published:** 2020-05-17

**Authors:** Ying Wang, Xiaoli Chen, Yun Yu, Yanqun Liu, Qing Zhang, Jinbing Bai

**Affiliations:** 1 School of Health Sciences, Wuhan University, Wuhan 430071, China; wangying9398@163.com (Y.W.); chenxl72@whu.edu.cn (X.C.); yuyun7169@163.com (Y.Y.); 2 Nell Hodgson Woodruff School of Nursing, Emory University, Atlanta, GA 30322, USA; jinbing.bai@emory.edu

**Keywords:** gut microbiota, infant, temperament, microbiota-gut-brain axis, Chinese birth cohort

## Abstract

Infant temperament characteristics play a critical role in children’s developmental pathways and can predict adulthood psychopathology. The diversity and composition of the gut microbiota are associated with human temperament in both adults and young children. However, the relationship between the gut microbiota and temperament in 12-month-old infants is rarely studied; this developmental period is when temperament reaches a relatively stable stage. We used high-throughput sequencing methods to explore whether temperament characteristics were associated with gut microbiota diversity and composition. Infants’ fecal samples were collected at 12 months of age for the gut microbiota analysis. Based on the primary caregivers’ reports, infants’ temperaments were measured using the Infant Behavior Questionnaire-revised (IBQ-R). This study included 51 infants, including 20 boys and 31 girls, with a mean age of 12.25 months. Results showed that soothability was positively correlated with maternal education level (β = 0.29, *p* = 0.043, adjust *p* = 0.025) and the abundance of *Bifidobacterium* genera (β = 0.62, *p* = 0.004, adjust *p* = 0.002). Conversely, cuddliness was negatively correlated with the abundance of *Hungatella* genera. There was no significant difference in temperament based on gender. This study demonstrated that gut microbiota composition was associated with temperament in 12-month-old infants. These results point to the importance of gut microbiota balance. Future studies on the mechanisms behind the gut microbiota affecting temperament are warranted.

## 1. Introduction

Studying temperament is an effective approach to study the differences in individual behavioral characteristics [1]. Based on the widely used Infant Behavior Questionnaire (IBQ) [1] and Infant Behavior Questionnaire-revised (IBQ-R) [2], cultural and sociodemographic modulatory factors could shape the development of temperament in early life. These factors include maternal race, maternal education level, caregivers’ psychosocial characteristics, family economic status, numbers of siblings, and feeding type [2,3,4,5]. However, infant temperament differences are hypothesized to be biologically based [6]. In studying infants’ temperaments, previous studies were restricted by infants’ age, sex, and birth order, and there was less focus on the exploration of the natural, biological mechanisms associated with temperament such as gut microbiota, immune factors, and inflammatory cytokines [2,7,8].

The bidirectional microbiota–gut–brain axis (MGB) connects the gut microbiota with the human brain, and this synergy in turn influences human behaviors [9,10]. The MGB axis [11] includes the central nervous system, the enteric nervous system, the autonomic nervous system, the neuro-endocrine (e.g., serotonin) and neuro-immune pathways (e.g., pro-inflammatory cytokines), and the gut microbiome [11,12]. The MGB pathway is proven to affect both the central nervous system and the hypothalamic–pituitary–adrenal axis (HPA); additionally, the MGB is proven to modulate multiple pathways such as neurotransmitters (e.g., serotonin), dopamine, and cytokines (e.g., interferon-γ and interleukin-6) [13]. In the growing field of MGB research, an increasing number of studies have found that emotional disturbances and social behaviors are associated with the dysbiosis of the gut microbiota. A randomized controlled human trial has shown that multispecies, probiotic consumption can reduce sadness and aggressive thoughts [14]; e.g., a treatment o*f Lactobacillus helvecticus R0052* and *Bifidobacterium longum R0175* in healthy people can significantly reduce anxiety and depression [15]. Similarly, treatment with *Bacteroides fragilis* of autism-like behaviors in mice improved behavioral defects including anxiety, compulsive marble burying, and ultrasonic vocalizations [16]. The gut microbiota is intricately associated with specific brain regions that are linked to emotional stimuli and cognition development [17]. Gao and colleagues [18] studied 12-month-old infants using functional Magnetic Resonance Imaging (fMRI) and found that gut microbiota diversity was associated with functional connectivity between the amygdala and thalamus; gut microbiota diversity was also associated with connectivity between the anterior cingulate cortex and anterior insula. These studies suggest a significant connection between gut microbiota and human mental states and emotions.

Evidence supports associations between the gut microbiota and temperament in both children and adults. In adult populations, Kim and Park initially proved that different temperament traits are associated with various enterotypes [19]. Additionally, in a study of 77 children aged 18–27 months old, Christian et al. found that *Rikenellaceae* genera were significantly associated with temperament in both boys and girls [20]. Coincidentally, Kim and Park have also suggested that temperament differences induced by the gut microbiota may occur in early life [19]. Temperament can be detected as early as 3 months of age and dynamically changes during the first year of life; however, a 12-month-old infant’s temperament will usually remain stable until early childhood [1,2]. Putnam and colleagues [21] found that considerable homotypic continuity existed in human temperament from infancy to 7 years of age. Infant gut microbiota developed rapidly due to the transition to an adult diet after the age of 12 months [22]. Thus, 12 months of age is an intriguing time point for us to study in order to explore the relationship between temperament and gut microbiota. However, whether gut microbiota diversity and composition are associated with temperament in infancy at the age of 12 months old or younger is rarely studied.

In this study, we hypothesized that (1) infants’ temperament characteristics are associated with the gut microbiota diversity and composition at age 12 months; and (2) the temperament differences with respect to the gut microbiota are independent of the infants’ gender, maternal education status, delivery mode, feeding type, and probiotic consumption. The purpose of this study was to examine 51 infants’ gut microbiota (using high-throughput sequencing) and temperament ratings (based on caregivers’ report) at 12 months old and to explore the relationships between these two variables.

## 2. Materials and Methods

### 2.1. Study Design, Setting, and Participants

This study enrolled 62 mother–infant dyads. Mothers during late pregnancy were recruited from an obstetric outpatient clinic in a Grade III A Hospital of Wuhan University in Wuhan, Hubei Province in China. All women were healthy. Infants were followed for 1 year after birth from November 2017 to February 2018. Because 11 of the families could not be reached by telephone or email at 12 months postpartum, 51 healthy infants were enrolled in the study. This study was approved by the Research Ethics Boards of Medical School of Wuhan University (Approval code: JKHL2017-03-03; Approval date: 03/03/2017). Informed consents were obtained from all the participants.

### 2.2. Data Collection

Every maternal participant completed three questionnaires face-to-face, including demographic characteristics (e.g., maternal age, education status, child’s sex), infants’ early life events (feeding type, probiotic consumption at age 6 months), and an assessment of infants’ temperament at an age of 12 months.

Our previous study [23] revealed that early life *Clostridium butyricum* consumption was associated with infants’ gut microbiota during later life. We included the probiotic consumption data for analysis in this study. The newborn consumption of probiotics containing *Clostridium butyricum* (*C. butyricum*, at least 75 × 10,000,000 CFU) was to facilitate defecation and prevent jaundice in early neonates (0–1 months after birth). In this study, 42 newborns were prophylactically given probiotics.

The Infants Behavior Questionnaire-Revised (IBQ-R) Chinese version was used to assess infants’ temperament at 12 months [2]. The IBQ-R was developed for infants aged 3–12 months old. This measure includes 14 dimensions of temperament that are subdivided into three composite scales: extraversion, negative affectivity, and orienting/regulation. This measure has good reliability and validity [2].

Infants’ fecal samples were collected at participants’ homes by a trained researcher; samples were taken from infants’ diapers according to the Human Microbiome Project (HMP) protocol [24]. Temperament questionnaires were completed at the time of sample collection. After collection, fecal samples were transported in a cooler (+4 °C, 1.5 h on average) to the laboratory and were frozen at −80 °C prior to further analysis. In total, 51 fecal samples were collected for high quality Illumina sequencing. Please refer to our previous study [23] for the processing of DNA extraction, polymerase chain reaction (PCR) amplification, sequencing, and data analysis.

### 2.3. Statistical Methods

We compared infant temperaments between boys and girls using the Mann–Whitney U test. The multivariate analysis of the general liner model (GLM) was used to analyze the significant factors of infant temperament. The Spearman correlation (denoted by r) was used to investigate the association between infant temperament and infant microbiota. We subsequently chose the relevant gut microbiota and prior predictors (e.g., infants’ sex and maternal education status) to identify potential associations in temperament using multiple linear regressions, controlling for delivery mode, feeding type, and probiotic consumption.

For the gut microbiota data analysis, the distribution of microbial community was visualized by Circos at the genus level. Principal coordinate analysis (PCoA) was conducted using the OTU information from each sample according to the Bray–Curtis distance matrix (gender: male vs. female, delivery mode: cesarean vs. vaginal, probiotic consumption: yes vs. no, and feeding type: breast vs. mixed). A permutational multivariate analysis of variance (PERMANOVA) was used to analyze the explaining degree of different grouping factors to the sample difference based on OTU level using Bray–Curtis distance matrix. A genus relative abundance ≥ 1% was selected for the abundance analysis.

The data for genera relative abundance were the centered log ratio (CLR) transformed before all analyses. We set the significance level at α = 0.05. Multiple hypothesis tests were adjusted using the Benjamini–Hochberg false discovery rate (FDR), and associations were considered significant below an FDR threshold of 0.05. These analyses were performed using SPSS version 21 (IBM, Chicago, IL, USA) and R [25].

## 3. Results

### 3.1. Participant Characteristics

This study included 51 infants (20 boys and 31 girls). These infants had a mean age of 12.3 (mean ± SD: 12.3 ± 0.25) months. In this study, all the mothers were of Han nationality; the mean maternal age at the time of delivery was 32.3 (Mean ± SD: 32.3 ± 4.51) years. All mothers were married (Table 1).

### 3.2. Infant Temperament at 12 Months Old

Mean (SD) scores of three IBQ-R dimensions were 5.11 (0.55) for extraversion, 3.86 (0.74) for negative affectivity, and 4.69 (0.66) for orienting/regulation. In addition, gender presented no differences in temperament ratings according to the Mann–Whitney U test (*p* = 0.18, *p* = 0.26, *p* = 0.62). Maternal education level (β = 0.13, CI = (0.07, 0.19), *p* = 0.043, adjust *p* = 0.025) was positively related to soothability.

### 3.3. Infant Gut Microbiota at 12 Months Old

In total, 2,541,440 usable sequences and 308 OTUs were obtained from these infants using Illumina MiSeq. Pan analysis indicated that only a small number of new shared phylotypes would be expected with additional sequencing. The value of Good’s coverage for all children was 99.6%.

**Figure 1:**
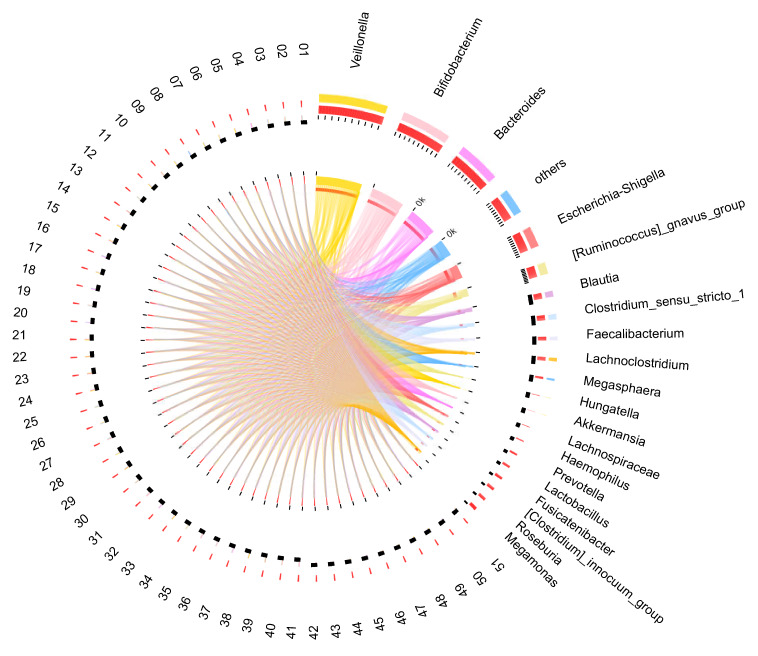
Distribution of microbial community for each sample at genus level. The data were visualized by Circos, and the width of the bars from each genus indicate the relative abundance of that genus in the sample.

The bacterial distribution was characterized in terms of the relative taxonomic abundances. A total of 10 phyla, 18 classes, 25 orders, 46 families, and 128 genera were obtained from 51 infants’ samples at 12 months of age. Figure 1 shows the taxonomic compositions of the dominant bacteria at the genus level. In terms of the relative abundance, 15 genera were above 1%, and the five most abundant genera were *Veillonella* (22.6%), *Bifidobacterium* (17.04%), *Bacteroides* (14.75%), *Escherichia-Shigella* (7.87%), and the *Ruminococcus gnavus* group (5.14%).

**Figure 2:**
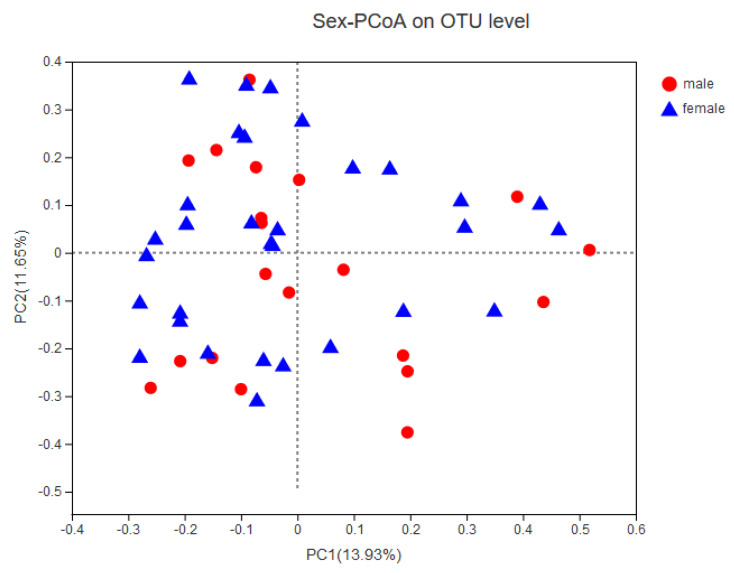

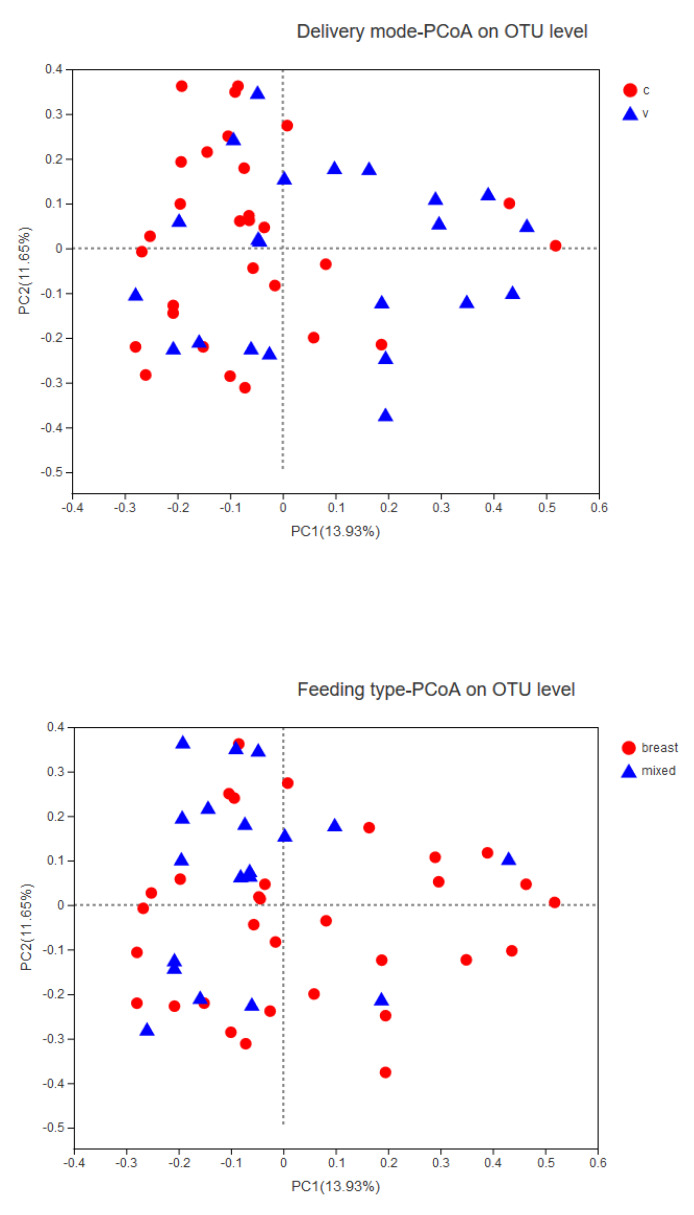

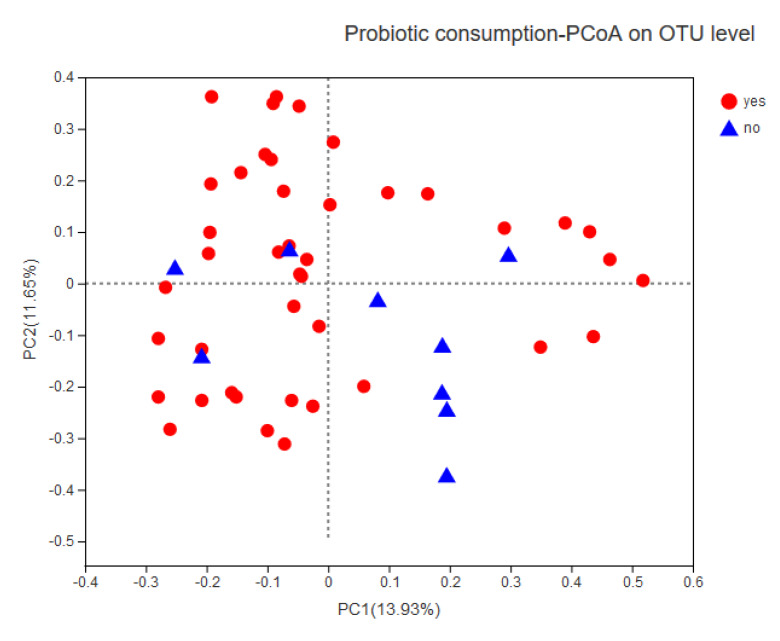
Principal coordinate analysis (PCoA) of infant gut microbiota using Bray–Curtis distance matrix. A two-dimensional PCoA was used to describe the relative abundance of infant gut microbiota based on sex, delivery mode, feeding type, and probiotic consumption. Each point represents a single sample and is color-coded into different groups.

Gender was not separated well by principal coordinate analysis (PCoA) based on the Bray–Curtis distance matrix at the OTU level (ANOSIM: R = −0.03, adjust *p* = 0.56, Figure 2); gender was also not separated by probiotic consumption (ANOSIM: R = −0.08, adjust *p* = 0.80), feeding type (ANOSIM: R = 0.05, adjust *p* = 0.15) or delivery mode (ANOSIM: R = 0.05, adjust *p* = 0.06).

### 3.4. Association between Infant Temperament and the Gut Microbiota

**Figure 3:**
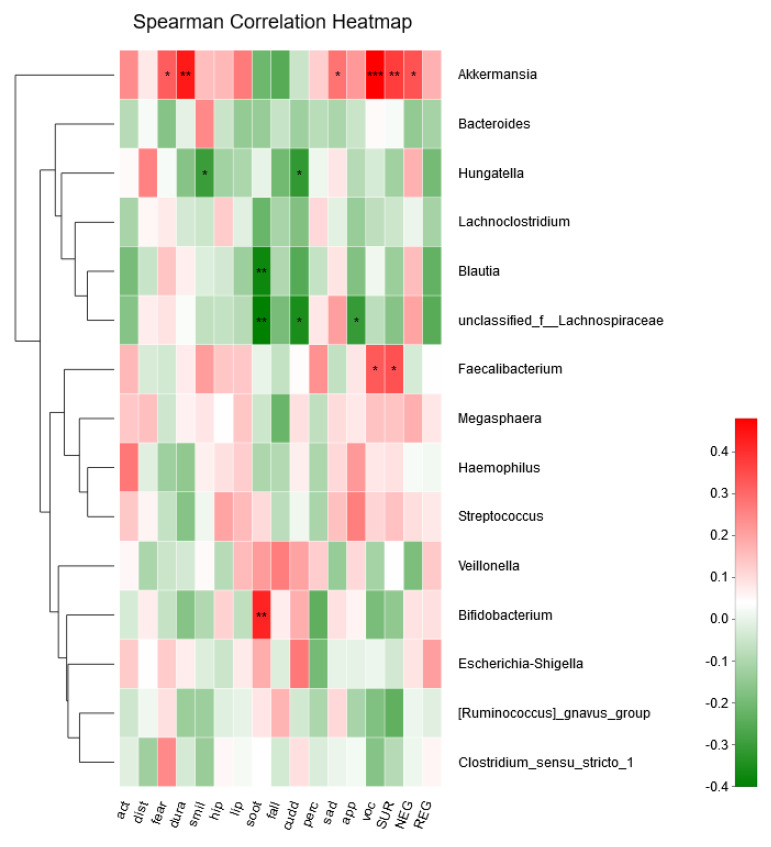
Relationships between bacterial composition and infants’ temperament. The figure shows a heatmap in which samples have been clustered according to their compositional profiles. Bacterial genera appear color-coded according to their under (green) or over-representation (red) in the samples (* 0.01 < *p* ≤ 0.05, ** 0.001 < *p* ≤ 0.01, *** *p* ≤ 0.001).

Among 14 dimensions and three composite scales of IBQ-R, six genera were associated with infant temperament (Figure 3). Multiple linear regressions were conducted when infant temperament was identified as the outcome, and both relevant genera and other potential confounding factors (e.g., delivery mode, feeding type, and probiotic consumption) were controlled in the model. The abundance of *Bifidobacterium* (β = 0.13, CI = (0.102, 0.158), *p* = 0.004, adjust *p* = 0.002) was positively related to soothability. Cuddliness was negatively correlated with abundance of *Hungatella* in infants (β = −2.78, CI = (−3.77, −1.79), *p* = 0.008, adjust *p* = 0. 028).

## 4. Discussion

Boys and girls showed no significant differences in infant temperament at 12 months old. This finding was consistent with previous studies [7,26,27]. Gartstein [7] explored the stability of infants’ temperament by gender in a sample of 315 children from South Korea, which was the only non-Western study, and found no differences in temperament between boys and girls. However, research in Western countries found that temperament ratings differed significantly by infant gender. The first study of gender differences in temperament was measured by IBQ-R in 2003 [2], and this study showed that female infants showed higher scores on the fear subscale and that male infants were rated higher on the activity and high-intensity pleasure subscales. Another study from the United States [20] found significant differences in temperament based on gender. Boys had higher ratings in high-intensity pleasure and motor activation, while girls reported higher ratings for inhibitory control and soothability. Specifically, compared to boys, girls were rated lower on the surgency/extraversion subscale, and girls were rated much higher on the effortful control subscale. Thus, the discrepancy in temperament differences between boys and girls in these various studies could be a result of cultural differences.

In this study, infant soothability in 12-month-olds increased with higher maternal education. Soothability refers to children’s reduction of fussing, crying, or distress when caregivers use soothing techniques [2]. In practice, caregivers’ soothing techniques are key factors that influence the development of greater soothability in older children. Furthermore, more highly educated mothers may have a more positive view of their parent–child interactions, thus leading to a higher level of intimacy and pleasure for both infants and mothers. Additionally, multiple studies have suggested that maternal education level contributes to positive parent–infant interaction by influencing parenting styles and timing of paternal investment. [28,29]. One study observing 139 Chinese infants aged 12–24 months found that highly educated mothers were more likely to utilize guiding behaviors, and they developed higher levels of intimacy with their infants compared to less-educated mothers [30]. When caring for their children, highly educated mothers tended to lead their children in playing or learning in a variety of ways. The ability to love and feel beloved among highly educated mothers was higher, and these mothers were more likely to perceive that they exerted a positive influence on their children through parenting. Although parenting styles and mothers’ responsiveness to children’s behaviors vary by culture [31], maternal education level is associated with infants’ physical health and psychological behaviors across different cultural contexts; this is consistent with the results of the present study. Behaviors and health outcomes associated with the mother’s education level include infants’ mental health problems [32], watching TV during meals [33], vocabulary acquisition [34], and cognitive and motor outcomes [35]. Overall, our findings indicated that infants’ temperaments were closely related to maternal education level.

This study found that high soothability was associated with a predominant relative abundance of *Bifidobacterium* in infants’ gut microbiota at 12 months old. Meanwhile, the temperament of cuddliness (ability to express enjoyment and the molding of one’s body to being held by caregivers) was negatively associated with an abundance of *Hungatella*. In accordance with our study results, previous studies have shown that the human gut microbiota is associated with temperament. In a study of 60 healthy Korean adults, Kim and Park were the first to promote the existence of temperament [19] differences between enterotypes 1 and 2 in adult populations. Compared to enterotype 2, enterotype 1 had significantly higher scores in novelty seeking and reward dependence behaviors. They suggested that differences in temperament and stability of temperament might be shaped by interactions with the gut microbiota during early life stages (e.g., infancy). Furthermore, one study of 77 children aged 18–27 months old [20] found that gut microbiota diversity and composition were associated with temperament as assessed by the Early Childhood Behavior Questionnaire (ECBQ) [36]; among these children, those with a greater phylogenetic diversity in their gut microbiome exhibited greater surgency/extraversion. Some gender differences were also found; in girls, Rikenellaceae were positively correlated with levels of fear, and in boys, sociability was positively correlated with the abundances of *Ruminococcaceae* phylum and *Parabacteroides*. Additionally, there were positive relationships between high-intensity pleasure and *Dialister*, Rikenellaceae phyla, as well as between activity level and *Dialister*, Rikenellaceae phyla. In agreement with these previous studies, our research further confirmed the possible effects of the gut microbiota on infant temperament at 12 months old.

*Bifidobacterium*, which accumulates in large amounts in the human gastrointestinal tract, is an integral part of the human gut microbiota. Multiple studies have verified its remarkable function in immune-modulation [37], obesity [38], infection [39], balance of gut microbiota [40], and especially in mental health disorders [41]. In fact, changes in emotional processing modulated by the gut microbiota have been established by a number of observational studies on probiotic consumption. One study in mice [42] suggested that *Bifidobacterium longum 1714* and *Bifidobacterium breve 1205* supplements were effective treatments for decreasing anxiety-like behaviors, and they were as effective as the anti-anxiety medication escitalopram. Additionally, a study in rats [43], using an electric shock model, found that consumption of *Bifidobacterium longum R0175* over a 30-day period led to a significant decrease in anxiety. In animal models of Alzheimer’s disease [44], it was observed that *Bifidobacterium breve* treatment may improve cognitive function and counteract some degree of impairment. In a human study, the consumption of *Bifidobacterium* was shown to effectively improve depression–anxiety conditions, increase defecation frequency, and decrease body mass index in healthy elderly people [45]. Similarly, supplementation with the prebiotic *Bifidobacterium longum 1714* resulted in an improvement of the frontal midline electroencephalographic mobility and hippocampus-dependent memory performance in healthy volunteers [46]. Together, these studies suggest that the human anxiety behaviors, depression behaviors, and cognitive function were altered by *Bifidobacterium* species. This is further supported by our results finding high soothability in infants to be associated with significantly dominant *Bifidobacterium* in their gut microbiome.

*Hungatella* is a functional genus that carries genes related to polysaccharide metabolism and is involved in the production of acetate and propionate. Numerous studies have proved that it is responsible for enhancing efficient energy harvesting and gastrointestinal mobility [47,48]. Additionally, in patients experiencing constipation, *Hungatella* was significantly more abundant compared to healthy controls [49]. Similarly, work in patients with Parkinson’s disease [50] also found that there was a significant positive correlation between the relative abundance of *Hungatella* and patients’ constipation, which was independent of their Parkinson’s disease. Meanwhile, there has been far less research on the association between mental behaviors and *Hungatella*. In an observational study of Chinese navy soldiers, it was found [51] that after a long voyage mission, the abundance of *Hungatella* in the gut microbiota of soldiers had significantly decreased. These findings opened exciting possibilities for exploring the relationship between an increased abundance of *Hungatella* and lower cuddliness in infants. It is important to explore the relationships between *Hungatella* and personality or temperament-related behaviors.

There were some limitations that reduced the generalizability of these study findings. The full study provided a small-scale test of associations between infants’ gut microbiota and temperament at the age of 12 months. Additionally, the majority of infants in our study were taking probiotics at the time of sample collection, which may affect the generalizability of the study. Our understanding of these connections could be strengthened by exploring the gut microbiota as it relates to temperament in older children. Future research should replicate this study with a larger sample, using a longitudinal study design to explore the link between infant temperament and gut microbiota and its impact on children’s later life. In addition, this study did not address the role of diet quantity or quality at the time of stool sample collection. Although parents fed their children according to standardized guidelines for infants’ diets, the potential mechanisms of food quantity may account for some of the differences in the associations.

## 5. Conclusions

This study examined the association between infants’ temperament and the gut microbiota at the age of 12 months. We found that soothability was positively correlated with the abundance of *Bifidobacterium* and maternal education level. There was a negative correlation between cuddliness and an abundance of *Hungatella*. This study contributed to our understanding of microbiota–behavior/physiology interactions and confirmed that this relationship is stable and robust by 12 months of age. It also provides a new approach to studying behavioral characteristics in teens, which should start in infancy. We should emphasize the intestinal health and gut microbiota balance before the transition to adult foods. The deficiencies of the study include infants’ probiotic consumption and the small sample size, which somewhat limits the study’s conclusions; consequently, further research in this area may be warranted. From the MGB viewpoint, this study was not designed to address detailed causal interaction pathways between the gut microbiota and temperament. Thus, future research should explore the possibility of incorporating metagenomics and metabolomics and analyzing these associations to understand the causes behind the appearance of this new evidence in a large infant cohort.

## Figures and Tables

**Table 1:** Demographic and birth characteristics (*N* = 51).

Items	N (%)/Median (IQR)
Maternal education	
<12 a	4 (7.8%)
12 a	31 (60.8%)
>12 a	16 (31.4%)
Sex	
Male	20 (39.2%)
Female	31 (60.8%)
Mode of delivery	
Caesarean	29 (56.9%)
Vaginal	22 (43.1%)
Feeding mode	
Breast	32 (62.7%)
Mixed	19 (37.3%)
Probiotic consumption	
Yes	42 (82.4%)
No	9 (17.6%)
